# How doctors record breaking bad news in ovarian cancer

**DOI:** 10.1038/sj.bjc.6600816

**Published:** 2003-03-18

**Authors:** J M Kirwan, D G Tincello, T Lavender, R E Kingston

**Affiliations:** 1 Liverpool Women's Hospital, Crown Street, Liverpool L8 7SS, UK; 2 Department of Obstetrics & Gynaecology, University of Leicester, Leicester General Hospital, Gwendolen Road, Leicester LE5 4PW, UK; 3 University of Central Lancashire, Preston PR1 2HE, UK

**Keywords:** ovarian cancer, documentation, records, diagnosis, collusion, age

## Abstract

Revealing the diagnosis of cancer to patients is a key event in their cancer journey. At present, there are no minimal legal recommendations for documenting such consultations. We reviewed the Hospital records of 359 patients with epithelial ovarian cancer in the Mersey Area between 1992 and 1994. We identified the following factors: age, hospital, postcode, surgeon, stage of disease and survival. These were compared to information recorded at the time of the interview such as person present, descriptive words used, prognosis, further treatment and emotional response. In 11.6%, there was no information recorded in the notes. The diagnosis was recorded in 304 (94.7%), prognosis in 66 (20.6%) and collusion with relatives in 33 (10.3%). A total of 42 separate words/phrases were identified relating to diagnosis; cancer was recorded in 60 (19.6%). Collusion was three times as common in the patients over 65 years (17.9 *vs* 5.7%, *P*=0.001). There was a reduction in the number of diagnostic words recorded in the patients over 65 years (90.3 *vs* 98.3%, *P*=0.002) and by type of surgeon (*P*=0.001). Information was often poorly recorded in the notes. We have shown that the quality of information varies according to patient age, surgeon and specialty.

A diagnosis of cancer can cause great suffering to patients and families. The revealing of the diagnosis of cancer to patients is a key event in their cancer journey. The word cancer is often avoided in these consultations (Thomsen *et al*, 1993). Over recent years, communication and information have increasingly been considered important in helping people with cancer (Fallowfield *et al*, 1994; Coulter, 1998). Research indicates that the vast majority of cancer patients want to be informed of their illness (Meredith *et al*, 1996). Women with ovarian cancer need honest communication that is appropriate to their level of understanding. Communication needs will vary across patient's age, stage of disease and treatment. At present, there are no minimal legal recommendations for documenting such consultations. Furthermore, complaints made by patients often focus on a perceived failure of communication rather than on clinical errors (Department of Health, 2000).

In its guide ‘Good Clinical Practice’ (General Medical Council, 2001), the General Medical Council (GMC) states ‘a good medical record should contain sufficient information to: identify the patient; support the diagnosis; justify the treatment; document the course and results and promote continuity of care among healthcare providers’, and continues, ‘Doctors must keep colleagues well informed when sharing the care of patients. Without good notes, this is impossible’.

Experts agree that improved communication between health professionals and cancer patients is essential for the delivery of high-quality care (Department of Health, 2000). As one in three people will be diagnosed with cancer during their lifetime, investigation of this influential consultation is vital to improving and monitoring the service we as clinicians provide. We report here an observational survey of the quality of information relating to giving the diagnosis recorded in the hospital case notes of patients with ovarian cancer.

## MATERIAL AND METHODS

We reviewed the Hospital records of patients diagnosed with epithelial ovarian cancer in the Mersey Area between 1992 and 1994. The appropriate authorisation for the study was obtained from the individual consultants in the Hospitals audited.

Information and recurrent themes relating to communications or interviews following the diagnosis of epithelial ovarian cancer were identified in the medical and nursing notes. The themes were: ‘diagnosis’ words, ‘prognosis’ words, further treatment, emotional response, patients' understanding, information-seeking behaviour and evidence of collusion. Descriptive words were recorded verbatim. Data extraction was performed by three of the authors independently (JMK, DGT and TL); any discrepancies were resolved in discussion. Collusion was defined as any entry recording discussion of the diagnosis or prognosis between relatives and medical or nursing staff where the patient was actively excluded.

The following demographic data were also extracted from the case notes: age, hospital, postcode, year of surgery, surgeon, stage of disease and debulking achieved, length of survival and preoperative suspicion of ovarian cancer. The International Federation of Gynaecology and Obstetrics (FIGO) stage (FIGO Cancer Committee, 1986) was obtained by review of the operation notes and histology by one of the authors (JMK). We calculated the underprivileged area score (UPAS) using the patients' postcode (Jarman, 1993).

Data were stored on a statistical software package for the social sciences (SPSS version 10, SPSS Inc., Chicago IL, USA). The frequency of recorded information and themes were compared to the demographic variables using *χ*^2^ test, Fisher's exact test, Mann–Whitney *U* test or Kruskall–Wallis as appropriate. Continuous data are presented as median (range) or mean (s.d.). Significance was set at 1%, taking into account Bonferroni corrections.

## RESULTS

We identified 359 patients with a histological diagnosis of epithelial ovarian cancer. There were 90 stage 1, 45 stage 2, 132 stage 3, 36 stage 4 patients and 56 patients where it was not possible to stage the disease. The mean age at diagnosis was 62.7 (13.6) years. A total of 331 patients underwent primary surgical debulking and 28 were diagnosed on either cytology of ascites and/or pleural fluid or a pelvic mass on imaging and a raised CA125. Follow-up to 5 years was available for all patients: 78.3% of patients survived 6 months, 55.1% 18 months and 29.1% 5 years. In 34 (9.5%) patients, there was no relevant information recorded in the case notes, and four patients were demented, so the following analyses are presented on the 321 patients where data were available. The diagnosis was recorded in 304 cases (94.7%), prognosis was documented in 66 cases (20.6%), emotional response in 103 (32.1%), patient understanding in 62 (19.3%), information seeking behaviour in 42 (13.1%), further treatment 216 (67.3%) and evidence of collusion with the relatives in 33 cases (10.3%).

Examples from the notes are shown below.

‘Findings discussed’
‘Pt informed of probable diagnosis of ovarian neoplasm, grateful for being informed’
‘Doctor thought patient was told of operation findings, pt speaks of cyst and that is all that was mentioned, Dr used words tumour, growth, but not malignant or cancer’
‘Patient does not want family to know her condition she will tell them in her own time’
‘Patient feels it has not registered with her what has happened’
‘Histology explained to patient and mum both state they understand’
‘Spoken with son and daughter told inoperable tumour they will discuss with their father and decide how much to tell their mother’

The person or persons present at the time of the consultation is show in Table 1

**Table 1:** Person(s) present

**Person(s) present**	***n***	**%**
No-one	127	39.6
Husband/partner	63	19.6
Generic ‘family’	27	8.4
Not recorded	26	8.1
Daughter	19	5.9
Other family	8	2.5
Son	7	2.2
Sister/brother	5	1.6
Parents	4	1.2
Friend, requested no family	2	0.6
Collusion^a^	33	10.3

a Patient actively excluded from discussion (see text).

. In over one-third of cases (39.6%), the patient was unaccompanied. The husband/partner was the commonest accompanying person (19.6%), who was often present with other members of the family (8.4%). Daughter(s) were more than twice as likely to be present than son(s). In one patient, the consultation was requested without the presence of the family. Collusion between family members and medical/nursing staff was documented in 33 cases (10.3%).

The diagnostic words and phrases recorded in the notes of the 304 patients are shown in Table 2

**Table 2:** Diagnostic words recorded in the 304 patients where there was an entry

**Diagnostic words recorded**	***n***	**%**
Operation	84	27.5
Diagnosis	79	25.8
Histology	68	22.2
Cancer	60	19.6
Findings	45	14.7
Tumour	35	11.4
Malignant	34	11.1
Cyst	12	3.9
Disease, mass	10	3.3
Carcinoma, situation	7	2.3
Results, procedure, lump, procedure and condition	4	1.3
Primary, growth and serious problem	3	1.0
Problem, abnormal cells, could not remove/separate, disease remains	2	0.7
Cytology, cells, looked odd, microscopic deposits, swelling, not simple, nasty, benign, secondaries, abnormal, neoplasm, tissue diagnosis, metastases, all removed, macroscopic clearance, suspicious, lesion and adherent	1	0.3

. A total of 42 separate words or phrases were identified relating to diagnosis, the commonest were generic words such as ‘operation’, ‘diagnosis’ and ‘histology’. In 60 patients (19.6%), the word cancer was specifically recorded. There were many words that occurred on three or fewer occasions; however, the vast majority were recorded in conjunction with other words in the table such as ‘operation’ and ‘diagnosis’. There were 42 cases of only one word being recorded.

Documentation of prognosis given to the patient at the time of the consultation was present in only 66 women (20.6%). For these women the words used are shown in Table 3

**Table 3:** Prognosis word recorded in 66 women where there was an entry

**Prognosis recorded**	***n***	**%**
Poor	16	15.2
Palliative	6	5.7
Extent of disease	4	3.8
Progression, TLC, good outlook, inoperable, unable to remove all	3	2.9
Spread of disease, guarded, cure impossible	2	1.9
Surgery enough, all being well should have response, uncertainty of response, cure unlikely, advanced, keep at bay, early stage, deterioration, likely prognosis, all away, should be fine, terminal, bleak, extremely poor, too far gone, nature take its course, no further treatment, bad, optimistic, untreatable	1	1.0

. There were 31 separate words or phrases used to describe prognosis. The commonest prognosis word recorded was ‘poor’, recorded on 16 occasions (15.2%). Further treatment was recorded in 216 patients (67.7%), and is detailed for these patients in Table 4

**Table 4:** Further treatment recorded in the 216 women where there was an entry

**Further treatment**	***n***	**%**
None	113	35.3
Chemotherapy	49	15.3
Further treatment	27	8.4
Undecided	12	3.7
Surgery	11	3.4
Palliative/hospice	3	0.9
Radiotherapy	1	0.3
		
Total	216	100

. In 113 patients (35.3%), no further treatment was recorded, and in 49 (15.3%) chemotherapy was indicated.

Practitioners recorded the response to diagnosis and/or prognosis on 137 patients. Thematic analysis revealed that these responses fell into one of three categories: emotional response, understanding and information-seeking behaviour.

The most commonly reported words recorded following initial diagnosis/prognosis were ‘anger’, ‘upset’ and ‘distressed’. However, there was a continuum of emotional responses recorded from ‘feeling positive’ and ‘indifference’ to ‘contemplating suicide’ and ‘wishing to die’. Patients were recorded as being ‘frightened’, ‘depressed’ and ‘abandoned’. Others were reported as being ‘in denial’ or ‘not unduly concerned’.

Practitioners recorded their perception of the level of understanding of the information provided. Words commonly used were ‘accepting’, ‘understands’ and ‘realistic’. Records describing the patients understanding were often followed by a brief description of a coping strategy, such as ‘patient going to battle on making disease fit round her life, ‘taking one day at a time’ or ‘making the most of the time she has left’.

One of the frequently reported reactions to the diagnosis/prognosis was to seek more information. A dichotomy of reactions was recorded. Records suggested that some women were not ready to receive further information at the initial session. Entries included ‘she does not want to talk about the future and what may happen’, ‘does not want to ask any more questions’ and ‘feels it unnecessary to discuss condition’. Other records highlighted the need to seek immediate information, such as ‘wanted to ask more questions’ and ‘asked to see oncologist’.

There were 145 women (45.2%) over 65 years of age, and 176 women (54.8%) under 65 years. There was a significant reduction in the number of diagnostic words recorded in the over 65's compared to the under 65's (90.3 *vs* 98.3%, *P*=0.002, Fisher's exact test). Collusion was more than three times as common in the over 65's (17.9 *vs* 5.7%, *P*=0.001, Fisher's exact test). Emotional response was more frequently recorded in the under 65's (38.1 *vs* 26.2%, *P*=0.024, *χ*^2^ test). There was no difference in prognosis, understanding or information-seeking behaviour recorded.

The overall 5-year survival was 29.1%. There was no relation between survival at 6 or 18 months and any themes, categories or variables.

The UPAS was calculated for each patient using her postcode. The median score was 3.68, range –99.00 to 62.00. There was no relation between UPAS and any themes, categories or variables.

In 56 of the 359 cases (15.6%), it was not possible to stage the patient because of poor note keeping. In these 56 patients, there was a significant reduction in the diagnostic words being recorded in this group compared to all other stages. A 26.8% had no information at all (*vs* 6.3%, *P*< 0.0001) and 41.1% had no diagnostic words (*vs* 9.9%, *P*=0.001).

A general gynaecologist operated on 177 patients (55.1%), a special interest gynaecologist 61 (19.0%), a general surgeon 28 (8.7%), an SpR in gynaecology in 37 (11.5%). A total of 18 patients (5.6%) were not operated on. The frequency with which diagnostic words were recorded in the notes differed significantly depending upon the surgeon who performed the operation: special interest gynaecologists 100%, SpR 97.3%, general gynaecologist 95.5% and general surgeon 89.3% (*P*=0.001, Fisher's exact test).

Collusion occurred significantly more often in women who did not have an operation compared to those who did (*P*=0.0001, *χ*^2^ test). There was no difference in the incidence of collusion according to type of surgeon.

## DISCUSSION

To our knowledge, this is the first study to specifically investigate information recorded in the hospital case notes following the diagnosis of ovarian cancer. These are unique data with demographic information, 5-year survival and serve to highlight several important issues in the management of ovarian cancer patients. Although data were obtained for this study from patients with epithelial ovarian cancer, the results are likely to be relevant to other cancer patients.

CancerBACUP (BACUP, 1996) and other cancer organisations recommend that patients have a member of family or a close friend present when bad news is given. In our study, over one-third of patients were often told essential information on their own. If a relative was present, it was most commonly the partner/husband. However, this is a group of women with a mean age of 62.7 years and a proportion will be single, divorced or separated or their partner may not be fit enough to travel to hospital. When children were present, daughters were more than twice as likely to be present than sons.

Most studies show that lay populations have a universal dread of cancer (Fallowfield, 1997). The word ‘cancer’ therefore, with all its connotations and meaning, is stressful for both patient and doctor alike. A doctor's failure to employ accurate terminology squanders an ideal opportunity to correct misconceptions about the disease. Furthermore, euphemisms such as lump, growth, serious problem (as recorded in Table 3) are confusing and unhelpful. Such euphemisms only serve to reinforce how awful the disease cancer really is as the doctor is unable to use the word ‘cancer’. In our study, 42 separate phrases were employed with the term ‘cancer’ only recorded in 18% of patients. Our study shows that a large proportion of medical staff still uses these ambiguous terms. Moreover, with an average reading age of 91/2 years (Department for Education and Employment, 1999), few patients would have fully understood the full meaning of many of these words.

Patients now want to know the truth (Meredith *et al*, 1996). Despite the advances in information available to patients, many doctors still unwittingly hurt their patients while trying to protect them by withholding information (Thomsen *et al*, 1993). However, few would state that they actively withhold the diagnosis of cancer from their patients. In our study, there was evidence of collusion in over 10% of patients. Active collusion was twice as common in the over 65's, a group who are more vulnerable as they are less likely to question the doctors' decisions (Nordin *et al*, 2001). This is despite evidence that the elderly not only want to be given the same information as the young (Ganz, 1997), but also want access to the same radical treatment and same chance of disease cure (Nordin *et al*, 2001).

General and special interest gynaecologists operated on the majority of patients in the study. There were significantly more diagnostic words recorded by special interest gynaecologists compared to general gynaecologists or general surgeons. This is to be expected, as they are more used to dealing with such patients and liaising with multidisciplinary teams.

We found that the UPAS had no bearing upon any of the information variables we studied. We found this surprising, as anecdotally it appears that patients from higher socioeconomic groups are given more information, partly because they seem better informed and ask more questions. This is confirmed by a review of 16 955 first-time enquirers accessing the CancerBACUP information service, where the users were predominantly middle class (Boudioni *et al*, 1999). It was therefore reassuring to see that all patients in our study were treated the same way.

The Royal College of Surgeons of England states that Surgeons must, ‘Ensure that a record is made by a member of the surgical team of important events and communications to the patient or supporter (for example, prognosis or potential complications) (The Royal College of Surgeons of England, 2002). It was therefore disappointing to find that in 9.5% of patients, there was no recording in the case notes of this consultation. Where a record was made, however, it was not possible to validate the accuracy of the information recorded in the hospital notes in this study. It is entirely possible that the quality of information given was in excess of that recorded in the case notes and that our data simply reflect poor note keeping. We have no way of confirming this. However, since many of the words recorded are evasive and lacking in clear meaning, we suspect that they are indeed a true reflection of the kind of language used with the patients during the consultation. Our data have revealed some fascinating but also disturbing trends, which can only be confirmed and fully examined by a prospective study of information giving, employing taped consultations and qualitative research methods.

**Figure 1:**
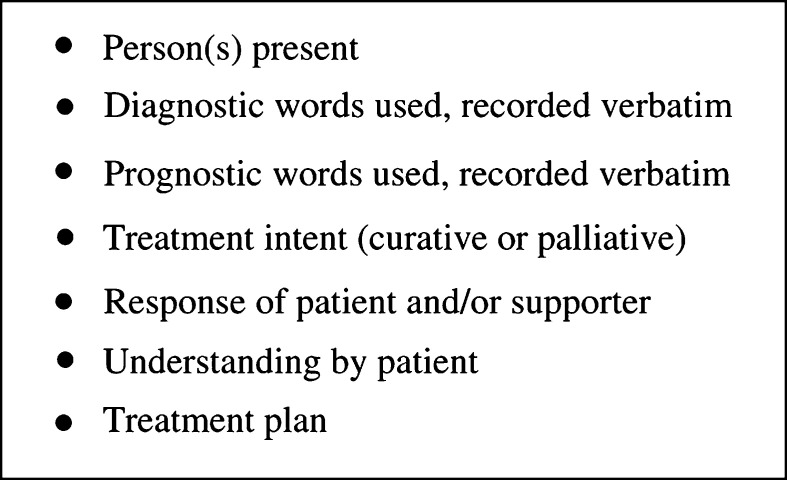
Suggested minimum data set for recording diagnostic conversations.

Effective clear communication has profound influences on both patients and health-care professionals. It influences the rate of recovery, pain relief and psychological well-being (Fallowfield *et al*, 1990; Stewart, 1996). Moreover, poor communication at the very start of a cancer journey can leave patients anxious, uncertain and generally dissatisfied with their cancer care (Audit Commission, 1993). Good communication skills can be taught, and in the cancer-care arena can be delivered in a variety of ways (Fallowfield *et al*, 1990; Wilkinson *et al*, 1999). As we move towards multidisciplinary team management in all stages of the disease trajectory (Expert Advisory Group on cancer to the chief medical officers of England and Wales, 1995), good communication between individual team members gains importance. Thus, clear written documentation of doctor–patient consultations, through diagnosis, treatment, relapse and terminal illness forms a vital key in total patient management. Our suggested minimum data set that should be recorded in the hospital notes following such a consultation is shown in Figure 1

. This recorded information would therefore enable clinicians and other professionals involved in the future care of a patient immediate access to what the patient understands, thus allowing them to build on this knowledge. This would also act as a clear record of the doctor–patient communication at that time in their cancer journey. This will be important in defending litigation cases or as an adjunct when discussing previous treatment with a patient and/or their supporter.

This study emphasises the importance of high-quality clinical practice coupled with good note keeping, and echoes the recommendations of the Bristol Royal Infirmary Inquiry for accurate note keeping and audio tape recording facilities when an important diagnosis, course of treatment or prognosis is being discussed (Bristol Royal Infirmary Inquiry, 2001).
